# Staff survey on using the new clinical risk assessment framework for teams (CRAFT) tool

**DOI:** 10.1192/bjo.2021.781

**Published:** 2021-06-18

**Authors:** Pamela Swift, Brian Gillatt, Emma Drysdale, Hollie Walker, Pamela Johnston, Emma Jackson

**Affiliations:** 1 Royal Alexandra Hospital; 2 Rowanbank Clinic

## Abstract

**Aims:**

Risk assessment and management are crucial elements of clinical practice in mental health. Healthcare Improvement Scotland identified risk management as a key area for change, with risk tools identified as one necessary component. In NHS Greater Glasgow and Clyde (GG&C) the CRAFT tool replaced the Glasgow Risk Screen (GRS) in October 2019. The CRAFT tool is a 2 page document that comprises a broad risk screen, details of historical risk events and prompts for family and carer involvement. The aim of this study was to assess staff attitudes to the CRAFT, 12 months after it had been rolled out. Looking at whether the CRAFT tool is used to inform decision making about risk in clinical settings and if patients were involved in the risk management process.

**Method:**

An electronic staff survey was distributed to all clinical staff within NHS GG&C Mental Health Services. Clinical staff includes the following professional groups: Medical, Nursing, Psychology, Occupational Therapists and Allied Health Professionals. Contact details were accessed via the relevant managers and surveys were sent via secure global address lists. Questions were focused around the following areas: time taken to complete/update/frequency of use/contact and ease of use, role in decision making, patient and carer involvement/knowledge, view on the impact of the CRAFT.

**Result:**

There were 209 responses. This represents a response rate of approximately 10%. 89% of respondents had completed a CRAFT tool at some point but only 38% had received training. 15% reported that the CRAFT did not aid decision making about risk in clinical settings, whereas 37% said it did and 42% said it did sometimes. 46% report patients are consulted most of the time (34%) or always (12%). The qualitative impression was that the CRAFT was an improvement on its predecessor. However common themes from responders highlighted a lack of clinical relevance or impact decision making, lack of training in filling it out and cumbersome integration with the electronic case notes.

**Conclusion:**

Staff perceptions of the CRAFT tool were generally negative with many feeling it was a box ticking exercise that had minimal real world impact on patient risk and its management. However many felt it was an improvement over the previous risk tool and the majority used it at some point to aid clinical decision making.

